# 

**Published:** 1915-05

**Authors:** 


The Package Library gives access to the best in $500.00 worth of medical journals

Vol. XXX MAY, 1915 No. 11

### TEXAS Medical Journal

### "Red Back"

ESTABLISHED JULY, 1885

PUBLISHED MONTHLY AT AUSTIN, TEXAS, BY

MRS. F. E. DANIEL, - - - Publisher and Managing Editor

Subscription, $1.00 a Year, in Advance. - - - - Single Copies, 10 Cents

Entered at the Postoffice at Austin, Texas, as second class mail matter.

